# The Impact of Absorbed Solvent on the Performance of Solid Polymer Electrolytes for Use in Solid-State Lithium Batteries

**DOI:** 10.1016/j.isci.2020.101597

**Published:** 2020-09-21

**Authors:** Gabrielle Foran, Denis Mankovsky, Nina Verdier, David Lepage, Arnaud Prébé, David Aymé-Perrot, Mickaël Dollé

**Affiliations:** 1 Département de Chimie, Université de Montréal, 1375 Avenue Thérèse-Lavoie-Roux, Montréal, Québec H2V 0B3, Canada; 2 Prospective Lab, Total SA, Paris La Défense 92069, France

**Keywords:** Electrochemical Energy Storage, Energy Storage, Mechanics of Materials, Polymers, Energy Materials

## Abstract

The effects of solvent absorption on the electrochemical and mechanical properties of polymer electrolytes for use in solid-state batteries have been measured by researchers since the 1980s. These studies have shown that small amounts of absorbed solvent may increase ion mobility and decrease crystallinity in these materials. Even though many polymers and lithium salts are hygroscopic, the solvent content of these materials is rarely reported. As ppm-level solvent content may have important consequences for the lithium conductivity and crystallinity of these electrolytes, more widespread reporting is recommended. Here we illustrate that ppm-level solvent content can significantly increase ion mobility, and therefore the reported performance, in solid polymer electrolytes. Additionally, the impact of absorbed solvents on other battery components has not been widely investigated in all-solid-state battery systems. Therefore, comparisons will be made with systems that use liquid electrolytes to better understand the consequences of absorbed solvents on electrode performance.

## Graphical Abstract

**Figure:**
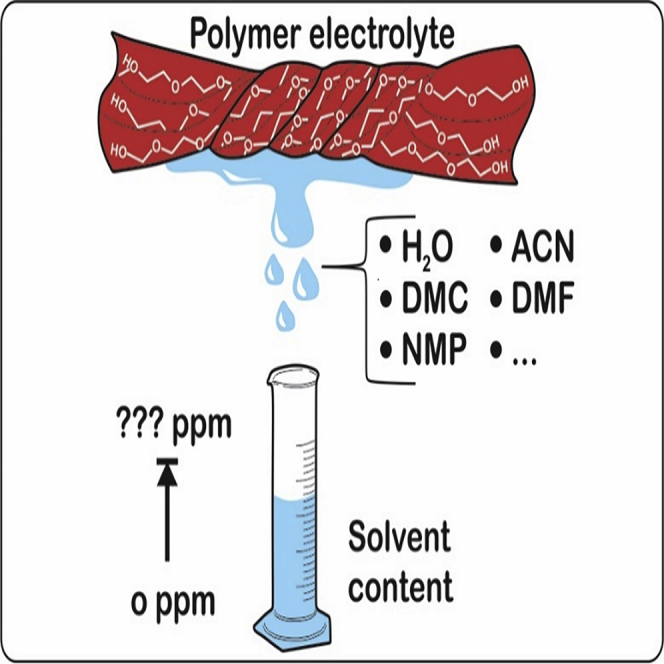

## Teaser

Many of the polymer electrolytes that are used in solid-state batteries readily absorb solvents. Although solvents have been shown to be difficult to completely remove from these materials following typical preparation procedures, solvent content tends to be under-reported. This review cautions that while absorbed solvents tend to increase the ionic conductivity and polymer crystallinity in solid polymer electrolytes, purposely adding them to electrolyte systems may decrease the performance of lithium-based electrodes.

## Introduction

**Figure 1:**
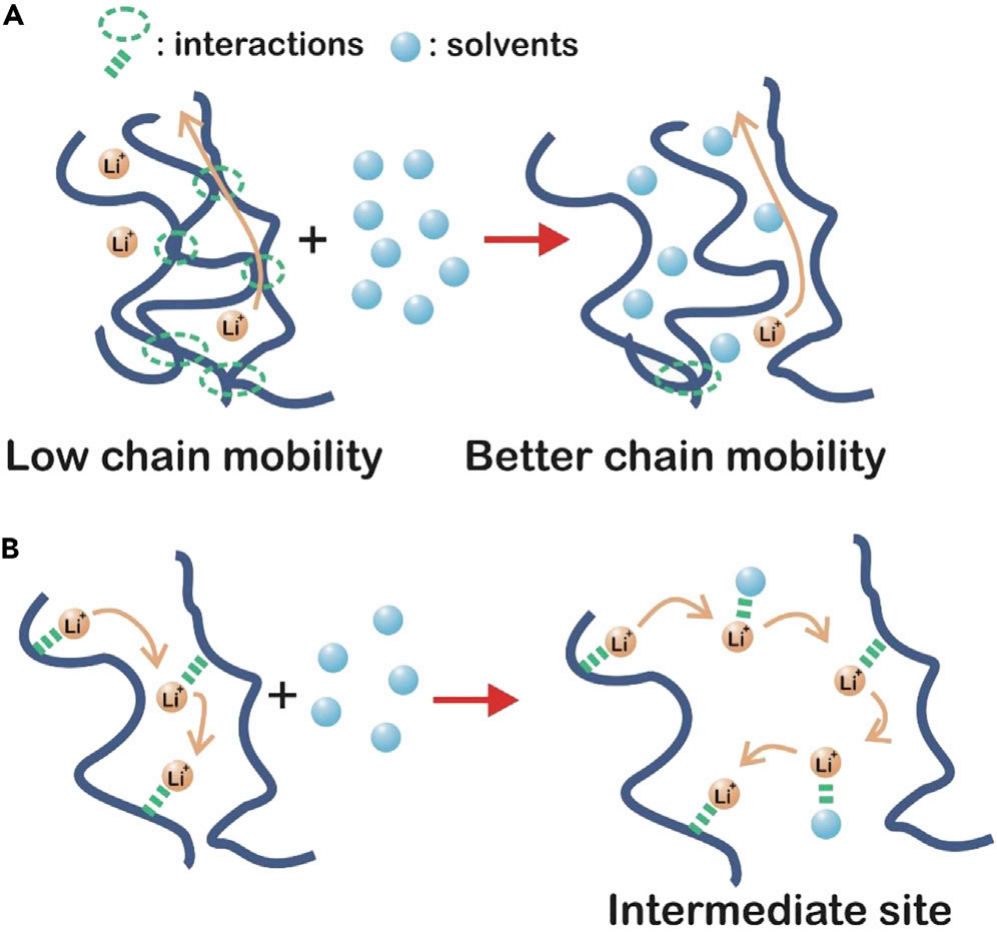
Structural Changes Caused by Solvent Absorption Impact Lithium Ion Mobility in Polymer Electrolytes (A) Ion mobility in a dry polymer can be impeded by interactions between polymer chains, and residual solvent can act as a plasticizer and improve the mobility of the polymer chains. (B) Ion mobility is increased in the presence of solvent as the lithium cations have greater affinity for polar solvents than for the polymer side chains.

Battery performance depends on the solubility and mobility of lithium cations in an electrolyte medium (Wegner, 2006). As a result of their low glass transition temperatures (T_g_), low dielectric constants, and high affinity for lithium cations, poly(ethylene oxide) (PEO) and other similar polymers doped with lithium salt are commonly used as electrolytes in solid-state battery assemblies (Mindemark et al., 2018). These materials are comprised of a semi-rigid backbone and amorphous ramified side chain segments (Wegner, 2006). As lithium conductivity is dependent on polymer mobility (Figure 1A) and the ability of these materials to solvate lithium cations (Figure 1B), the electrochemical and physical properties of this system under a variety of conditions (e.g., temperature, salt content, co-polymerization) have been investigated since the 1980s (Wegner, 2006). This review is focused on the impact of solvent absorption in solid polymer electrolytes for use in all-solid-state battery systems.

Solvent content has remained largely under-reported for the past four decades in polymer electrolytes for all-solid-state battery systems. The lack of information surrounding the solvent content of these materials is not ideal as polymer electrolytes are often prepared via solvent casting, which, like the absorption of water during atmospheric exposure or the absorption of other solvents during glovebox storage, provides a medium through which the dry polymer, and associated lithium salt, can absorb solvents such as acetonitrile and dimethylformamide (DMF) (Bhattacharja et al., 1986; Sun et al., 2015; Andrews et al., 2018; Ohno et al., 2020). Limited reporting on solvent content in polymers is likely the result of a combination of the fact that most polymers absorb solvents to some degree and that proper drying is difficult (Sepe, 2014). Proper drying requires attention to the temperature, moisture content, and the flow rate of air across the polymer surface (Sepe, 2014). Additionally, the drying temperature should ideally remain below T_g_, resulting in a complex set of conditions that must be maintained to appropriately dry a given polymer (Sepe, 2014).

Previously performed characterizations of solvent-containing polymer electrolytes have shown that increased solvent content typically causes cation solvation as a result of the lithium cations having more affinity for the solvent than for the ether oxygens located on the PEO segments (Fullerton-Shirey and Maranas, 2009). Cation solvation has been typically associated with increased cation mobility and increased ionic conductivity (Armstrong and Clarke, 1984b). In addition to affecting the transport properties of solid polymer electrolytes, adsorbed solvents also tend to impact polymer structure. Water and other solvents have been found to have a plasticizing effect on PEO and other commonly used polymers, resulting in lower T_g_ values for amorphous polymer fractions, decreased crystallinity, and increased side chain mobility (Harris and Rukavina, 1995; Zhang et al., 2018). Overall, the impact of ppm-level amounts of adsorbed solvent on solid polymer electrolytes has been shown to be significant (Commarieu et al., 2019).

In addition to impacting electrolyte performance, absorbed solvents (particularly water) are expected to significantly impact the stability of lithium anodes in all-solid-state battery systems. Solvents are generally believed to decrease anodic stability as a result of reactions with the anode surface (Homann et al., 2020). However, as suggested by Homann et al. (2020), some of these issues may result from deficiencies in commonly used methods to measure the electrochemical stability of battery systems, such as linear sweep voltammetry, when they are applied to systems with polymer electrolytes (Fu et al., 2005). Due to the experimental complexities associated with quantifying electrochemical failure in all-solid-state batteries being outside of the scope of this work, the electrode section of this review focuses on comparing the electrochemical properties of batteries based on their solvent content along with a discussion of how increased solvent content can contribute to battery failure.

Despite the potential for significant impact on battery performance, the fact that water and other solvents can usually be assumed to be present in these systems often goes unreported. Additionally, in instances where the presence of absorbed solvents is acknowledged, making comparisons between studies can be difficult as a result of differences in reporting methods: relative humidity, weight percent of water/solvent, water-to-ether oxygen ratios, and ppm of solvent. Therefore, this review will discuss the impact of water (section 2), acetonitrile (section 3), DMF (section 4), and other solvents (section 5) on the ion transport and structural properties of solid polymer electrolytes and attempt to discuss the impact of the presence of these solvents on electrode performance (section 6). As information regarding the impact of solvents on electrodes in all-solid-state batteries is not widely available, data from systems that employ liquid electrolytes will be reviewed under the assumption that electrodes would react similarly to absorbed solvents in solid polymer electrolytes. As similar electrode assemblies are used in all-solid-state systems, conclusions from these examples are likely also relevant for all-solid-state systems. Although solvents have been found to disrupt solid-electrolyte interphase (SEI) formation, the reaction between lithium metal and solvents has already been covered by many reviews (Cheng et al., 2015, Cheng et al., 2017; Peled and Menkin, 2017; Wang et al., 2018). The lack of information on the effects of solvents in electrolytes on battery performance suggests that solvent content should be more widely measured in these systems.

## Impact of Water Absorption on the Properties of Polymer Electrolytes

### Impact of Absorbed Water on Ion Mobility

Solvent casting is a commonly used procedure for the preparation of polymer electrolytes. This, coupled with the fact that the polymers themselves and the lithium salts which they are doped with are hygroscopic, has the potential to result in significant water absorption during membrane production (Andrews et al., 2018). PEO is known to interact with water via the formation of hydrogen bonds (Dormidontova, 2002). Although hydrogen bonding between water molecules is more energetically favorable than bonding to PEO, a molar fraction of roughly 0.2 has been predicted to hydrogen bond to PEO in the temperature range that is relevant to the preparation and use of PEO-based electrolytes (Dormidontova, 2002). The interaction between PEO and water causes polymer solvation which results in free lithium ions and likely contributes to increased ion mobility in hydrated systems (Hakem et al., 2006).

For example, Tanzella et al. (1981) have shown that PEO films doped with lithium bis(trifluoromethanesulfonyl)imide (LiTFSI) readily absorb water upon exposure to atmospheric conditions. Water absorption in this study was confirmed via the presence of O-H stretching vibrations in the infrared (IR) spectrum and a 3% mass loss between room temperature and 90°C in as-prepared samples recorded via thermogravimetric analysis (TGA) that was not observed in pre-dried samples (Tanzella et al., 1981). In addition to containing experimentally measurable quantities of water, the as-prepared sample exhibited higher ionic conductivity than the dried sample (Tanzella et al., 1981). This correlates with data obtained by Tominaga et al. (2009) who found that wet PEO-based polymer blends tended to be at least an order of magnitude more conductive than blends that had been dried prior to analysis.

In addition to being present in measurable quantities following synthesis and/or brief atmospheric exposure, there is evidence suggesting that absorbed water can be difficult to remove from polymer electrolytes. Work by Armstrong and Clarke (1984a) shows that significant drying may be needed to mitigate the effects of water absorption in polymer films. They prepared poly(ethylene adipate) films doped with LiTFSI by solvent casting under nitrogen atmosphere (Armstrong and Clarke, 1984a). Drying the samples at 100°C overnight was not sufficient to remove all of the water (Armstrong and Clarke, 1984a). This was observed when the ionic conductivity changed between heating and cooling cycles in a sample that was dried at 100°C but not in a sample that was dried at 140°C (Armstrong and Clarke, 1984a). The high desorption temperature suggests that water might be brought into the electrolyte via complexation with the lithium salt during the casting process, thus stabilizing the water inside the membrane at higher temperatures.

**Figure 2:**
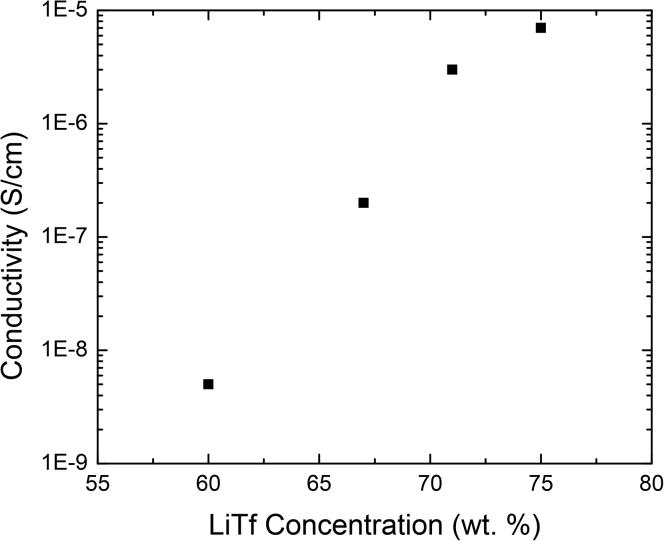
Ionic Conductivity of a Dry-Processed PAN Electrolyte as a Function of LiTf Content Ionic conductivity at 65°C as a function of LiTf concentration in a PAN film prepared by dry processing. Reprinted with permission from Forsyth et al. Journal of Polymer Science, Part B: Polymer Physics Copywrite 2000 Wiley and Sons(Forsyth et al., 2000a, 2000b).

As a result of lithium salts being hygroscopic, even polymer electrolytes that have been prepared via dry mixing have been shown to contain water. This was demonstrated by Forsyth et al. (2000a, 2000b) who prepared PAN-LiTf (lithium trifluoromethanesulfonate) electrolytes via dry mixing under nitrogen atmosphere followed by hot pressing at 150 to 180°C. These samples were additionally dried under vacuum for a period of three days (Forsyth et al., 2000a, 2000b). It was noted that increasing salt content resulted in significant increases in ionic conductivity (Figure 2). At 65°C, electrochemical impedance spectroscopy (EIS) measurements revealed ionic conductivity on the order of 10^−9^ S/cm in a sample that contained 65 wt% LiTf and on the order of 10^−6^ S/cm in a sample that contained 75 wt% LiTf (Forsyth et al., 2000a, 2000b). These results were interpreted to suggest that water was entering the electrolyte via complexation with the lithium salt. To verify this hypothesis, water uptake measurements were performed via gravimetric analysis at 50% relative humidity. Water uptake was found to increase with increasing salt content as the PAN sample containing 35% LiTf by weight had a water content of 3%, whereas the sample containing 65% LiTf by weight had a water content of 6% (Forsyth et al., 2000a, 2000b).

The preceding examples discuss systems where polymer electrolytes became hydrated as a result of absorption from atmosphere or following solvent casting. The facility with which these systems absorb water suggests that measurements to determine the water content of solid polymer electrolytes should be more widely performed. This is of importance as absorbed water has been shown to increase ionic conductivity, a desired electrolyte property. Quantifying water absorption and understanding its impact on ionic conductivity is an essential step in elucidating lithium conduction mechanisms in these materials (Mankovsky et al., 2020).

In order to purposely study the impact of hydration on ionic conductivity in solid polymer electrolytes, some researchers have studied systems where water was deliberately added. Deliberate water addition can be done in two ways: polymer electrolytes are exposed to a humid environment for a set time or controlled quantities of water are added under dry conditions. Examples of deliberate water addition to polymer electrolytes can be found in the work of Donoso et al. (1995) and Johansson et al. (1995) who exposed lithium salt doped PEO to D_2_O in order to track changes in cation mobility using nuclear magnetic resonance (NMR) spectroscopy. In both cases, sample D_2_O content was determined by mass. Donoso et al. (1995) performed ^7^Li NMR spectroscopy on PEO samples that were doped with either LiBF_4_ or LiClO_4_. Increasing D_2_O content was found to result in increased lithium cation mobility, which was demonstrated by decreasing T_1_ minimum temperature with increasing hydration between no added water and 20 D_2_O added per LiBF_4_ formula unit (or 86% by weight) (Donoso et al., 1995). T_1_ as a function of sample temperature was used to calculate activation energy. It was found that increasing D_2_O content decreased activation energy for lithium transport from 0.29 eV with no added water to 0.16 eV at 86 wt% water (Donoso et al., 1995). ^7^Li NMR was also used to measure the full width half maximum (FWHM) of peaks corresponding to LiTf in the polymer. FWHM was observed to decrease with increasing hydration from 10 kHz in the dry sample to 0.08 kHz in the D_2_O doped sample (Donoso et al., 1995). These changes were attributed to D_2_O acting as a plasticizer causing the free volume of the polymer chains to expand resulting in them becoming more mobile and exhibiting improved LiTf transport capability (Donoso et al., 1995).

**Figure 3:**
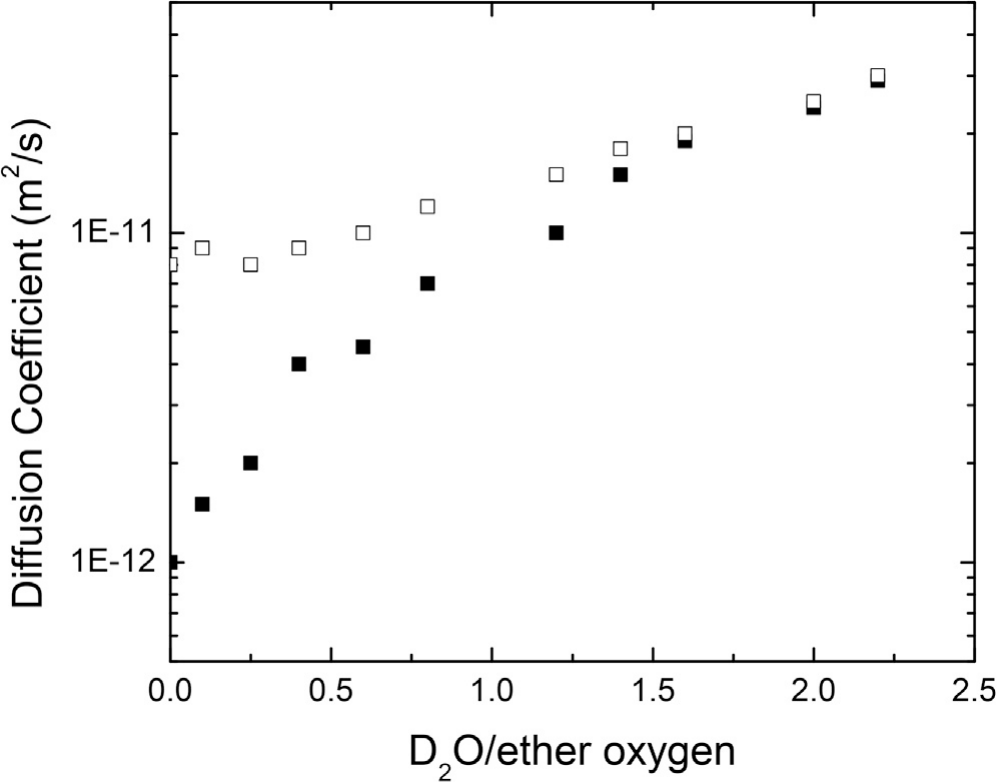
Lithium Self-Diffusion Coefficient for LiTf in PEO as a Function of D_2_O to Ether Oxygen Ratio Self-diffusion coefficients for free LiTf (□) and LiTf bound to PEO (▪) as a function of the D_2_O to ether oxygen unit ratio as measured by ^7^Li PFG NMR. Reprinted with permission from Johansson et al. Journal of Physical Chemistry Copywrite 1995 American Chemical Society(Johansson et al., 1995).

Johansson et al. (1995) performed pulse field gradient (PFG) ^7^Li NMR experiments on hydrated PEO samples that were doped with LiTf. Electrolyte hydration was found to result in an increase in the lithium cation self-diffusion coefficient for both lithium bound to the PEO polymer chain and the free LiTf (Figure 3) (Johansson et al., 1995). As water content was increased from 0 to 2.5 D_2_O per ether oxygen unit, the self-diffusion coefficients for both lithium environments increased and the difference between the self-diffusion coefficients of lithium on the polymer chain and the free salt decreased (Figure 3) (Johansson et al., 1995). Increased lithium cation mobility was attributed to solvation by water in both cases (Johansson et al., 1995). Solvation was particularly effective at increasing the mobility of lithium cations in the polymer chain as water is a better solvent for LiTf than PEO as a result of it having a higher dielectric constant (Johansson et al., 1995). Additionally, as PEO is hygroscopic, it was expected to absorb D_2_O and become less crystalline. Absorbed solvents are expected to act as plasticizers and modify the structure of the crystalline phase impacting chain mobility (Rubin and Andrews, 1968).

**Figure 4:**
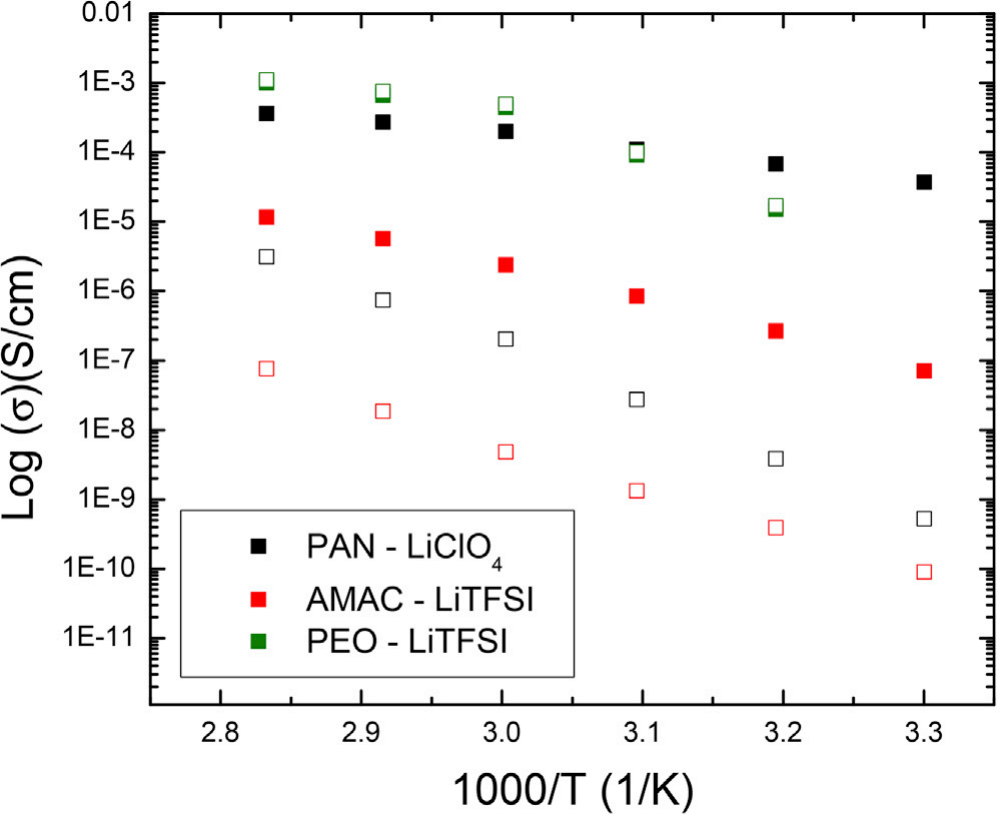
Differences in Ionic Conductivity in As-prepared Polymer Electrolytes and those that Have Been Dried Twice Comparison of ionic conductivity in as-prepared (▪) and twice dried (□) samples of PAN-LiClO_4_, AMAC-LiTFSI and PEO-LiTFSI electrolytes. Prepared based on data presented by Mankovsky et al.(Mankovsky et al., 2020)

Water content in three different polymer electrolytes—PEO-LiTFSI, PAN-LiClO_4_, and acrylonitrile methyl acrylate co-polymer (AMAC)-LiTFSI—was quantified by Mankovsky et al. (2020) using a water-specific moisture analyzer. The AMAC-LiTFSI and PAN-LiClO_4_ electrolytes were prepared via solvent casting, and the PEO-LiTFSI electrolyte was prepared without solvents using an internal mixer (Mankovsky et al., 2020). A significant finding in this study was that PAN-LiClO_4_ samples, which were dried for two 24-hr periods at 120°C under vacuum in a glovebox, experienced an increase in water content from 400 ppm to 7300 ppm after a 10-s exposure to ambient conditions (Mankovsky et al., 2020). In addition, as-prepared samples that were dried for one 24-hr period at 120°C (which is the typical polymer electrolyte preparation procedure) were found to contain significantly more water than samples that were dried twice. In the aforementioned PAN-LiClO_4_ sample, the dried twice sample contained 423 lithium ions per water molecule as opposed to 11 lithium ions per water molecule in the as-prepared sample. Similar observations were made for the PEO-LiTFSI and AMAC-LiTFSI electrolytes which contained 12 and 11 lithium ions per water molecule, respectively, in the as-prepared state and 106 and 102 lithium ions per water molecule, respectively, after being dried twice (Mankovsky et al., 2020). In addition to revealing how easily several thousands of ppm of water can be absorbed onto polymer electrolytes during typical preparation procedures or during a few seconds of exposure to ambient conditions, increasing water content is also attributed to increased ionic conductivity. At 30°C, the as-prepared polyacrylonitrile (PAN)- and AMAC-based polymer electrolytes are, respectively, about five and three orders of magnitude more conductive than the samples that were dried twice, showing the potential role of absorbed water in ion transport (Figure 4) (Mankovsky et al., 2020). For the PEO sample, similar ionic conductivities are observed in the as-prepared and dried twice samples (Figure 4) (Mankovsky et al., 2020). This could result from both samples being “relatively” solvent-free since the as-prepared sample was made via internal mixing.

Ma et al. (2006) studied the effects of increasing water content on glycerol-plasticized thermoplastic corn starch polymers that were doped with potassium, sodium, and lithium chlorides. Although glycerin (which was added as a plasticizer) was already present, additional increases in polymer chain mobility were obtained by exposing these polymers to water. The starch polymers were exposed to relative humidity of 0–100% at 20°C resulting in samples that contained 0.05 to 0.55 wt% water (Ma et al., 2006). Ionic conductivity was found to increase by up to five orders of magnitude with increasing water content depending on the type and quantity of salt that was present. Increased ionic conductivity was attributed to a combination of water weakening bonds in the starch causing the side chains to be more mobile and increasing the rate of salt dissociation and making the effective ion numbers higher (Ma et al., 2006). These effects were more prominent at lower salt concentrations because coordination with alkali salts tends to make polymer side chains more rigid.

As was stated above, water addition is expected to increase polymer chain mobility by acting as a plasticizer (Donoso et al., 1995; Johansson et al., 1995). While cation mobility is expected to increase with plasticization, changes in the polymer structure must be confirmed by measuring sample crystallinity. The next section explores how differential scanning calorimetry (DSC) and x-ray-based techniques have been used to explore changes in polymer electrolyte structure that have been caused by hydration.

### Impact of Water Absorption on Polymer Structure

Water is expected to have a plasticizing effect on solid polymer electrolytes resulting in increased mobility, flexibility, and free volume in the polymer chain (Donoso et al., 1995). As lithium cations have significant affinity for the polymer, it is anticipated that changes in polymer mobility will influence ion conductivity in these systems. Several authors have investigated the impact of water absorption on polymer thermal transitions by measuring T_g_ as a function of hydration (Donoso et al., 1995; MacFarlane et al., 1998; Sun et al., 2014; Zhang et al., 2015). The T_g_ can be defined as the temperature range over which amorphous regions of a polymer transition from being hard and brittle to being soft and flexible. Increasing water absorption has been shown to lower T_g_ (Donoso et al., 1995). Lower T_g_ values are expected to result in the polymer electrolyte becoming more mobile over a larger temperature range contributing to increased ionic conductivity (MacFarlane et al., 1998; Commarieu et al., 2019). In addition to changes in T_g_, the melting point (T_m_), where the crystalline regions of the polymer transition into a disordered melt, is lowered with increasing water content.

In addition to changes in T_g_, some authors have used x-ray-based techniques to observe the effects of hydration on polymer crystallinity. Zhang et al. (2018) used wide-angle X-ray diffraction to study crystallinity in LiTFSI- and LiClO_4_-doped PEO samples. The samples were exposed to environments with relative humidity between 0 and 55% (Zhang et al., 2018). It was found that polymer crystallinity decreased with increasing humidity when the relative humidity was greater than 10% (Zhang et al., 2018). These changes were coupled with a decrease in T_m_ and were attributed to lithium cation solvation by water weakening the strength of the bond between the cation and the polymer chains (Zhang et al., 2018). Water-salt interactions were found to be stronger than both water-polymer and polymer-salt interactions for this system (Zhang et al., 2018).

In addition to decreasing the strength of the bond between the lithium salt and the polymer, interactions with water have been shown to change the mechanism of salt complexation in solid polymer electrolytes. This phenomenon was demonstrated by Yang and Huo (2013) who doped PEO films with MgCl_2_ and its hydrated analogs: MgCl_2_^.^4H_2_O and MgCl_2_^.^6H_2_O. X-ray diffraction experiments showed that the incorporation of crystalline water, via hydrated MgCl_2_, changed how the PEO film complexed with the magnesium salt (Yang and Huo, 2013). Polymer-salt complexation was dependent on the quantity of crystalline water in the system as the incorporation of MgCl_2_ and MgCl_2_^.^4H_2_O resulted in a loss of crystallinity with increasing salt loading, whereas doping with increasing quantities of MgCl_2_^.^6H_2_O resulted in the formation of a new crystalline structure (Yang and Huo, 2013). As water absorption by the salt has been shown to impact complexation between the polymer and salt components of the electrolyte, more widespread measurement of water content would be beneficial for better understanding the relationship between salt loading and electrolyte crystallinity.

## Impact of Acetonitrile Absorption on the Properties of Polymer Electrolytes

Acetonitrile is commonly used as a solvent in the preparation of solid polymer electrolytes by the solution casting method (Armstrong and Clarke, 1984a; Sun et al., 2014; Commarieu et al., 2019). Like water, PEO can absorb acetonitrile which has been shown to result in changes in ionic conductivity and sample crystallinity by Commarieu et al. (2019). To this end, Weston and Steele (1982) specifically investigated the influence of the purity of the acetonitrile used for the solvent casting preparation method on the properties of the resultant polymer electrolytes. PEO-LiClO_4_ electrolytes were prepared by the solvent casting method in both high-purity and low-purity acetonitrile (Weston and Steele, 1982). The sample that was prepared in high-purity acetonitrile was found to have higher ionic conductivity, a lower activation energy for ionic transport (determined based on EIS data), lower crystallinity, and a lower T_g_ as determined by DSC (Weston and Steele, 1982). These differences in polymer properties lead to an investigation of differences between the low-purity and the high-purity acetonitrile samples. Further analysis of the acetonitrile samples by IR spectroscopy revealed that the higher purity sample contained more water than the low-purity sample (Weston and Steele, 1982). The water contained in the acetonitrile is believed to have been integrated into the polymer electrolyte via absorption by the lithium salt (Weston and Steele, 1982).

Hakem et al. (2006) have demonstrated that acetonitrile interacts with PEO differently than water does. As mentioned above, water can bind to PEO via hydrogen bonding interactions. This reduces the fraction of lithium ions that are bound to the polymer chain leaving yielding a higher fraction of mobile lithium (Dormidontova, 2002; Hakem et al., 2006). However, acetonitrile absorption tends to cause lithium ions to coordinate to the ether oxygen groups on the polymer resulting in charging and stretching of the polymer chains and decreased free ion motion (Hakem et al., 2006). These observations support Weston and Steele's work which showed that the sample that was prepared in acetonitrile with a higher water content had a higher ionic conductivity and lower T_g_.

The possibility of hygroscopic lithium salt acting as a vector for the incorporation of acetonitrile in the polymer electrolyte has been addressed by Sun et al. (2014) and Commarieu et al. (2019). Sun et al. (2014) observed that increasing LiTFSI content in a poly(trimethylene carbonate)-LiTFSI electrolyte resulted in increased ionic conductivity and decreased T_g_. As increasing salt content has been associated with decreased polymer motion as a result of hydrogen bonding in the absence of hydration, it is assumed that the observed solvation effects are a result of absorption of acetonitrile by the lithium salt during the solvent casting process (MacFarlane et al., 1998). Confirmation of the ability of hygroscopic lithium salt to absorb acetonitrile during solvent casting was provided by Commarieu et al. (2019) who dried PEC-LiTFSI electrolytes that were cast in acetonitrile at 60°C or at 100°C. Residual acetonitrile was observed in the sample that was dried at 60°C but not in the sample that was dried at 100°C (Commarieu et al., 2019). Although the intensity of the NMR signal corresponding to acetonitrile increased with increasing LiTFSI content, quantitative measurements of acetonitrile content were not provided in this study (Commarieu et al., 2019). Consequently, the sample that was dried at 60°C was more conductive (Commarieu et al., 2019).

Work by Łasińska et al., 2015 shows that absorbed acetonitrile can increase ion mobility by increasing the affinity of lithium cations for the polymer electrolyte. Co-polymers consisting of acrylonitrile and butyl acetate (poly(AN-co-BuA)) doped with LiTFSI were prepared by solvent casting (Łasińska et al., 2015). These electrolytes exhibited higher ionic conductivities and lower T_g_ than the parent polymer materials (Łasińska et al., 2015). These properties were enhanced with increasing LiTFSI addition (Łasińska et al., 2015). However, following aging under argon, ionic conductivity was observed to decrease while T_g_ was shown to increase (Łasińska et al., 2015). This behavior, which was found to be correlated to a decrease in the fraction of lithium cations that are bound to the polymer via Raman spectroscopy, was attributed to the evaporation of acetonitrile (Łasińska et al., 2015). Acetonitrile evaporation caused the affinity of the salt for the polymer to decrease by increasing the fraction of lithium salt that exists in an agglomerated state. This reflects the importance of quantifying the remaining solvents for the construction of salt-polymer phase diagrams, as well as to report unambiguously the optimum polymer/salt ratio with the highest ionic conductivity.

## Impact of Absorbed Dimethylformamide on Polymer Electrolytes

Like water, absorbed DMF has been shown to facilitate cationic motion in polymer electrolytes. DMF is commonly used when preparing PAN-based electrolytes via the solvent casting method. However, as a result of its high boiling point (150°C), DMF is very difficult to remove from polymer electrolytes despite the application of significant drying procedures (Liu et al., 2017). The nitrile groups contained within PAN make the polymer very polar and allow it to interact strongly with polar solvents via dipole-dipole interactions (Wu et al., 2012). The strength of these interactions, which depend on both the polarity and the structure of the solvent, is predicted to impact the ion conducting and structural properties of the resultant polymer electrolytes (Wu et al., 2012). This work discusses PAN containing absorbed DMF (this section), DMSO, and propylene carbonate (PC) (both in section 5). Based on the polarity and molecular structure of the solvents, the relative strength of these solvent-polymer interactions is expected to be DMSO > PC > DMF (Wu et al., 2012).

PAN films doped with LLTO (Li_0.33_La_0.57_TiO_3_) nanowires and LiClO_4_ were prepared via solvent casting in DMF (Liu et al., 2017). Despite drying at 120°C for up to one week, DMF was observed in the polymer electrolyte via IR spectroscopy and TGA (Liu et al., 2017). The TGA experiments showed that the polymer electrolytes contained about 3% DMF as a result of mass loss at 150°C (Liu et al., 2017). However, the effects of DMF on ionic conductivity were not discussed in this publication as the addition of active ceramic nanowires was the main focus of the work.

Chen-Yang et al. (2002) prepared PAN films doped with LiClO_4_ and Al_2_O_3_, an inert ceramic filler, via solvent casting in DMF. The samples were dried under vacuum for at least 24 hr but were still found to contain about 10% DMF as determined via TGA (Chen-Yang et al., 2002). X-ray diffraction and DSC studies showed that the addition of LiClO_4_ caused the material to become increasingly amorphous (Chen-Yang et al., 2002). Increased ionic conductivity with increased salt content suggests that these changes result in lithium ions becoming more mobile (Chen-Yang et al., 2002). As the addition of ceramic filler alone does not increase amorphousness (T_g_ actually increases), it is anticipated that it is the lithium salt that serves as a reservoir of absorbed DMF which can both decrease lithium cation affinity for the polymer and cause the polymer side chains to become more amorphous.

DMF is commonly used to prepare polyvinylidene difluoride (PVDF)-based polymer electrolytes via the solvent casting method. Following this process, DMF has been shown to be absorbed inside the polymer where it functions as a plasticizer resulting in increased ionic conductivity (Zhou et al., 2020). Zhou et al. prepared a series of PDVF-LiTFSI electrolytes with DMF and three other solvents: dimethylsulfoxide (DMSO), N-methyl-2-pyrrolidone (NMP), and dimethylacetamide (DMA) to quantify the effects of these trapped solvents on ionic conductivity and electrochemical stability relative to DMF (Zhou et al., 2020). TGA experiments showed that these electrolytes experienced extensive decomposition around 300°C (Zhou et al., 2020). This was attributed to the decomposition of the polymer electrolyte. However, mass loss below 300°C was attributed to the evaporation of absorbed solvents (Zhou et al., 2020). The mass percent of absorbed solvents (relative to the total polymer mass) in these samples following drying at 60°C and at 80°C for various time periods, determined based on the TGA data, is presented in Table 1.

**Table 1:** Solvent Content of PVDF-LiTFSI Electrolytes following Drying at 60 and 80°C (Data from the Journal of the Electrochemical Society, 2020) (Zhou et al., 2020)

Solvent	60°C, 1 day (wt%)	60°C, 10 days (wt%)	80°C, 1 day (wt%)	80°C, 4 days (wt%)
DMA	33.4	31.5	26.8	23.7
DMSO	25.0	25.0	16.7	17.1
NMP	28.2	28.1	23.7	22.3
DMF	19.2	18	15	15

**Figure 5:**
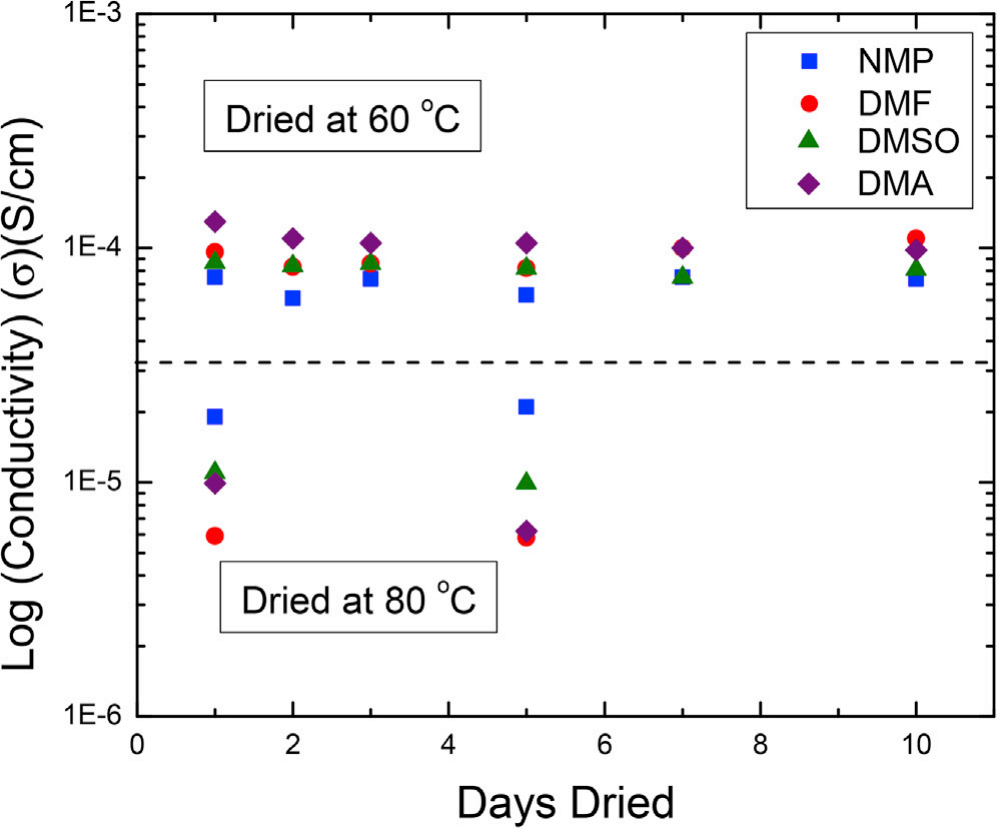
Impact of Solvent Type, Drying Temperature, and Drying Time on Ionic Conductivity in PVDF-LiTFSI Films Ionic conductivity of PVDF-LiTFSI films prepared with DMA, DMSO, NMP, and DMF after drying at 60 and 80°C for several days. Adapted from © 2020 Zhou et al.(Zhou et al., 2020) Published on behalf of the Electrochemical Society by IOP Publishing Limited. Reproduced with permission of IOP Publishing. All rights reserved.

Solvent content was somewhat correlated with ionic conductivity in these samples as drying at 80°C resulted in lower conductivities than drying at 60°C for all electrolytes (Figure 5). Also, drying for additional days at each temperature did not result in significant changes in either ionic conductivity of solvent content (Zhou et al., 2020). However, the absorbed solvent content did not correlate with the order of conductivities of the individual electrolytes (Figure 5). The DMA-containing electrolyte, which contained the most solvent at both drying temperatures, only had the highest conductivity after drying at 60°C (Zhou et al., 2020). The DMF-containing electrolyte, which contained the least amount of solvent at both drying temperatures, was the second most conductive sample after drying at 60°C (Zhou et al., 2020).

In addition to the effects of solvent absorption on ionic conductivity, the impact of solvent absorption on the stability of the lithium anode to cycling was also investigated. This was analyzed by cycling a Li-Li symmetric cell at a current density of 0.2 mAcm^−2^ and an area capacity of 0.1 mAhcm^−2^ (Zhou et al., 2020). The cells with electrolytes that were cast in DMF and DMSO and dried at 60°C were stable following 50 hr of cycling whereas the electrolyte that was cast in DMA and dried at 60°C lost stability after 46 hr and the electrolyte that was cast in NMP lost stability after 15 hr (Zhou et al., 2020). The differences in stability were attributed to the structural morphology of the resultant films as scanning electron microscopy showed that the films cast in DMF and DMSO had smooth surfaces, which decreases contact between the solvent and the electrode surface (Zhou et al., 2020).

## Impact of Other Absorbed Solvents on Polymer Electrolytes

NMP is a commonly used solvent for the preparation of polymer electrolytes by the solvent casting method (Kimura et al., 2015; Chintapalli et al., 2016; Łatoszyńska et al., 2017). As this procedure typically involves drying the resultant electrolytes for several hours both in air and under vacuum, the impact of NMP absorption on electrolyte properties has had limited discussion. Chintapalli et al. (2016) prepared PEO films containing LiTFSI by solvent casting in NMP. These films were dried at 90°C for 72 hr in air and then for 72 hr under vacuum prior to use (Chintapalli et al., 2016). NMP could not be detected in the resultant films via ^1^H NMR (Chintapalli et al., 2016). These findings suggest that commonly performed drying procedures may be more effective at removing NMP from polymer electrolytes than some of the drying procedures that are presented above for water, acetonitrile, and DMF. These findings suggest the PEO and lithium salts may have a lower affinity for NMP than other common solvents.

Even though a combination of air drying and vacuum drying may effectively remove NMP from polymer electrolytes, it is still worth exploring potential impacts of NMP absorption on ion mobility in these systems. This was investigated by using both DMF and NMP as plasticizers in a PVDF-based electrolyte for use in supercapacitors (Łatoszyńska et al., 2017). DMF was found to have a more significant impact on ionic conductivity in the electrolyte than NMP with a conductivity of 3.16x10^−4^ S/cm for the DMF-treated sample and 1.26x10^−5^ S/cm for the NMP-treated sample (Łatoszyńska et al., 2017). The lesser effect of NMP on ionic conductivity was attributed to the higher viscosity and the lower dielectric constant of the solvent (Łatoszyńska et al., 2017). The relatively low dielectric constant may account for DMF being more thoroughly removed from polymer electrolytes than other solvents.

DMSO can be used as a solvent to prepare polymer electrolytes via the solvent casting method. Voigt and Van Wüllen (2012) prepared PAN-LiBF_4_ films via solvent casting in DMSO. Although drying under vacuum at 90°C was performed to remove DMSO, ^1^H NMR showed that samples contained 9 to 50 mol% DMSO (Voigt and Van Wüllen, 2012). The quantity of residual DMSO in the samples depended only on the salt content with the LiBF_4_ to DMSO ratio being in the range of 1 to 1-2 for all samples, regardless of PAN content (Voigt and Van Wüllen, 2012). As ^7^Li linewidth experiments and ^1^H-^13^C cross polarization experiments showed that PAN does not participate in ion transport, all lithium-ion transport was attributed to the motion of lithium ions through the residual DMSO (Voigt and Van Wüllen, 2012). This could be a result of the high affinity of PAN for DMSO increasing the fraction of free lithium ions. Therefore, as was observed for water and acetonitrile, steps should be taken to determine and report DMSO content in samples that were prepared via casting in this medium.

In addition to preparation via solvent casting, solvents can also be added to polymer electrolytes directly as plasticizers. The use of ethylene carbonate (EC), PC, and succinonitrile (SN) as plasticizers will be discussed here. These additives have been shown to enhance ion mobility by decreasing the affinity of the lithium salt for the polymer (Wang et al., 2002; Kao et al., 2006). Wang et al. (2002) showed that preparing a PAN-LiTFSI electrolyte by solvent casting in PC resulted in decreased affinity between the salt and the polymer as a result of increased interaction between LiTFSI and PC via Raman spectroscopy (Wang et al., 2001, 2002). The high affinity of lithium salts for PC was also demonstrated by Kao et al. who solvated a Jeffamine triblock co-polymer cross-linked to a silicate network in a solution of PC and EC (Kao et al., 2006). Polymers containing more lithium salt were found to contain a higher weight percentage of the solvent (60% at lower salt content compared to 75% at higher salt content) (Kao et al., 2006). The plasticizer blend was found to increase ion dissociation as was demonstrated by 7Li NMR. Above −60°C, where the amorphous regions of the polymer are mobile, ^7^Li NMR shows that ion exchange occurs primarily between lithium ions in the plasticizer and lithium ions in the plasticized region of the polymer (Kao et al., 2006). ^7^Li signal intensity reveals that, in this temperature regime, most of the lithium is located in the plasticizer with no lithium being found in the immobile silicate network (Kao et al., 2006). Below this temperature range, the polymer crystallizes and the lithium salt is located preferentially in the rigid regions of the polymer that are strongly correlated to the silica network (Kao et al., 2006)^.^ These findings suggest that the role of the EC-PC solvent on ionic mobility is temperature dependent.

**Figure 6:**
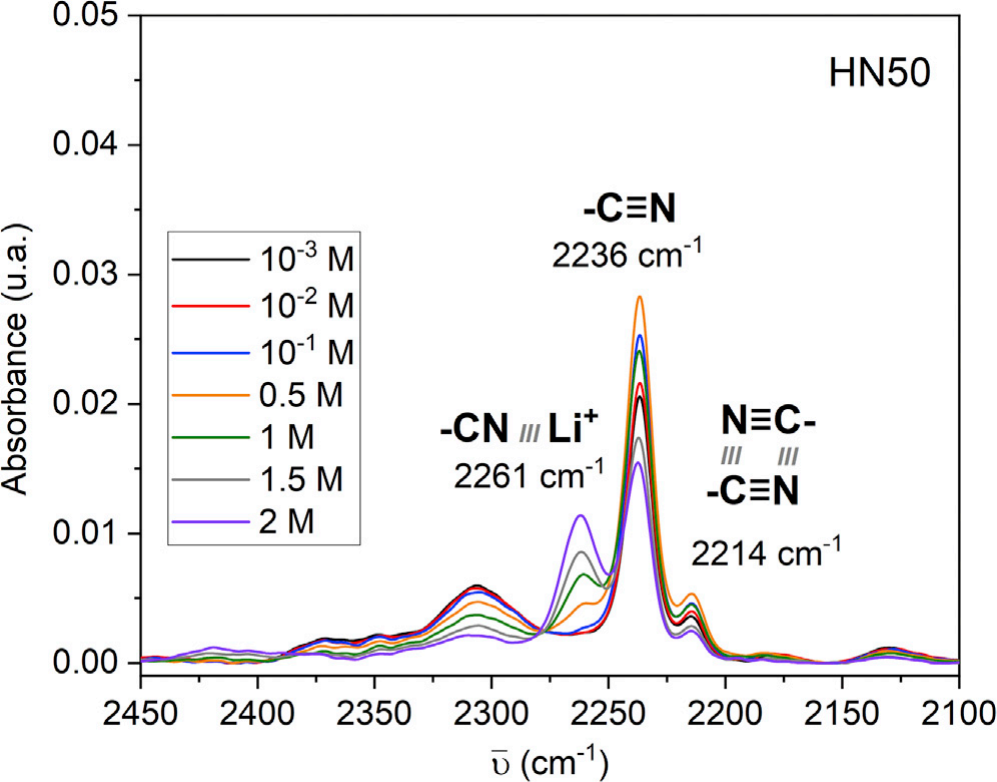
IR Spectrum of and HNBR Gel Electrolyte with Absorbed PC-LiTFSI IR spectrum of an HNBR gel electrolyte containing absorbed PC-LiTFSI. The peak at 2261 cm^−1^ indicates coordination between the nitrile group on the polymer and lithium ions. Reprinted with permission from Verdier et al. ACS Applied Energy Materials(Verdier et al., 2020) Copywrite 2020 American Chemical Society.

Verdier et al. (2020) compared the lithium-ion solubilizing ability of PC with that of DMC and DMSO in hydrogenated butadiene rubber (HNBR)-based gel electrolyte. This study used IR spectroscopy to monitor the intensity of the peak at 2261 cm^−1^, attributable to the interaction between nitrile groups and lithium ions, as a function of the concentration LiTFSI in the gel electrolyte (Wang et al., 1996; Verdier et al., 2020). In the sample containing PC, the peak at 2264 cm^−1^was observed at LiTFSI concentrations between 0.5 and 2 M (Figure 6) (Verdier et al., 2020). In the sample containing DMC, this peak was observed at LiTFSI concentrations between 10^−3^ and 2 M, whereas no interaction between lithium ions and the nitrile groups was observed in the sample that was swelled with DMSO (Verdier et al., 2020). This suggests that DMSO has a lower affinity for the HNBR-based electrolyte. The electrolyte that contained 2 M DMSO proved to be the least conductive at 30°C (2.2x10^−6^ S/cm as opposed to 8.1x10^−5^ S/cm for the PC-doped electrolyte and 1.8x10^−4^ S/cm for the DMF-doped electrolyte) as a result of less DMSO being trapped in the film when compared to the other solvents (Verdier et al., 2020).

SN was also shown to act as a plasticizer in PEO-LiBF4 electrolytes as SN addition decreased the tendency of these electrolytes to crystallize when salt content was high (Voigt and Van Wüllen, 2014). Sample crystallinity, as measured by X-ray diffraction and 7Li linewidth, decreased when the molar proportion of SN was greater than the molar proportion of PEO (Voigt and Van Wüllen, 2014). The addition of SN was shown to increase lithium mobility by acting as a solid lubricant and enhancing the mobility of the polymer chains. This effect was maintained down to temperatures as low as −30°C where increased ^7^Li line broadening and stronger dipolar coupling interactions showed that the crystallization of both the regular PEO and SN-plasticized phases occurred separately, limiting ion mobility (Voigt and Van Wüllen, 2014).

## Impact of Solvent Absorption on Solid-Sate Batteries

The preceding sections have focused on the effects of solvent absorption on the ion transport and morphological properties of solid polymer electrolytes for use in all-solid-state batteries. Many of these examples showed that water and/or acetonitrile absorption improved ion mobility through ion solvation and/or decreased polymer crystallinity. As these characteristics are typically associated with good ion transport properties in polymer electrolytes, it can be tempting to assume that hydrated electrolyte membranes should be installed in batteries to enhance the performance of these devices. However, as significant solvent content is not generally recommended for lithium battery systems, the impact of hydration on electrolyte and the electrode stability will be discussed in this section.

The stability of the electrolyte components to hydration will be explored here using PEO doped with LiTFSI as a model system. Even though one type of polymer and one type of salt are discussed, it is anticipated that the general conclusions can apply to other hygroscopic polymer and lithium salt assemblies that are used as electrolytes in all-solid-state batteries such as PAN (Yushkin et al., 2018). PEO doped with lithium salt is commonly used as an electrolyte material in all-solid-state batteries. PEO is considered stable in water as water does not cause the degradation of the material. However, PEO is completely soluble in water as a result of strong hydrogen bonding interactions between water and ether oxygen groups in the polymer (Husken and Gaymans, 2008). As discussed above, adsorbed water can act as a plasticizing agent in PEO by increasing the free volume in the polymer chains. This results in increased segment mobility, lower T_g_, and lower T_m_. While these characteristics have been shown to be favorable for ion mobility, significant water absorption by the polymer membrane can be damaging in a full battery assembly (Husken and Gaymans, 2008). This is notably because hydrophilic polymers, such as PEO, can experience significant swelling in the presence of water which can potentially result in rupturing the battery assembly (Husken and Gaymans, 2008).

Like PEO, LiTFSI, a lithium salt that is commonly used in polymer electrolytes, is also hydroscopic. As was described for PEO, this is not necessarily a problem for LiTFSI as the hydrated form is stable under both acidic and basic conditions (Huttner et al., 2020). However, the presence of water has been shown to have consequences on the performance of LiTFSI-based ionic liquids which are commonly used as electrolytes in lithium-ion batteries. Experiments performed by Kerr et al. (2018) on a series of phosphonium-based LiTFSI-doped ionic liquids suggest that increasing lithium content may actually reduce the negative impact of water exposure on other parts of the battery. When the lithium-to-phosphorous ratio was 1:1, cyclic voltammetry experiments showed that lithium deposition rate and cycling efficiency increased with increasing water content when water between 0 and 2000 ppm was present (Kerr et al., 2018). Above this threshold, electrochemical performance declined. The water content at which the maximum electrochemical efficiency was reached was only 500 ppm when the lithium-to-phosphorous ratio was 1:2 (Kerr et al., 2018). It is anticipated that the protecting effect of LiTFSI was a result of either lithium ions reducing the electrochemical activity of water or the lithium ions promoting the formation of a denser SEI layer (Kerr et al., 2018). However, despite the potential protection effects of lithium salts, significant water absorption by the electrolyte could be a problem for other components of the battery that are water-sensitive as the electrolyte layer is in constant contact with the electrodes (Husken and Gaymans, 2008). The next section will discuss the impact of hydration on the electrodes.

As many processing steps in battery manufacturing provide opportunities for water absorption by electrode materials to occur, Huttner et al. (2020) evaluated the mechanical and electrochemical properties of a graphite anode and a LiNi_0.6_Co_0.2_Mn_0.2_O_2_ (NMC) cathode following four post-drying schemes: no drying, drying under argon, and drying under vacuum for short (18 hr) or long (96 hr) periods of time. The Karl Fischer titration method was used to measure the water content of an anode-Celgard separator-cathode assembly with LiPF_6_ liquid electrolyte following each post-drying method (Huttner et al., 2020). The system had a total water content of 1417 ppm without post-drying, a total water content of 326 ppm after drying under argon, a total water content of 225 ppm after drying under vacuum for 18 hr, and a total water content of 136 ppm even after drying under vacuum for 96 hr (Huttner et al., 2020). It is worth noting that in this example, drying under argon reduced water content by 77% compared with no post-drying, whereas short- and long-term vacuum drying reduced water content by 84% and 90%, respectively. This suggests that electrode materials are less hygroscopic than polymer electrolytes as work by Mankovsky et al. (2020) showed that vacuum-dried (48 hr) PAN-LiTFSI contained 400 ppm water which increased to 7300 ppm after a 10 s exposure to ambient atmosphere.

**Figure 7:**
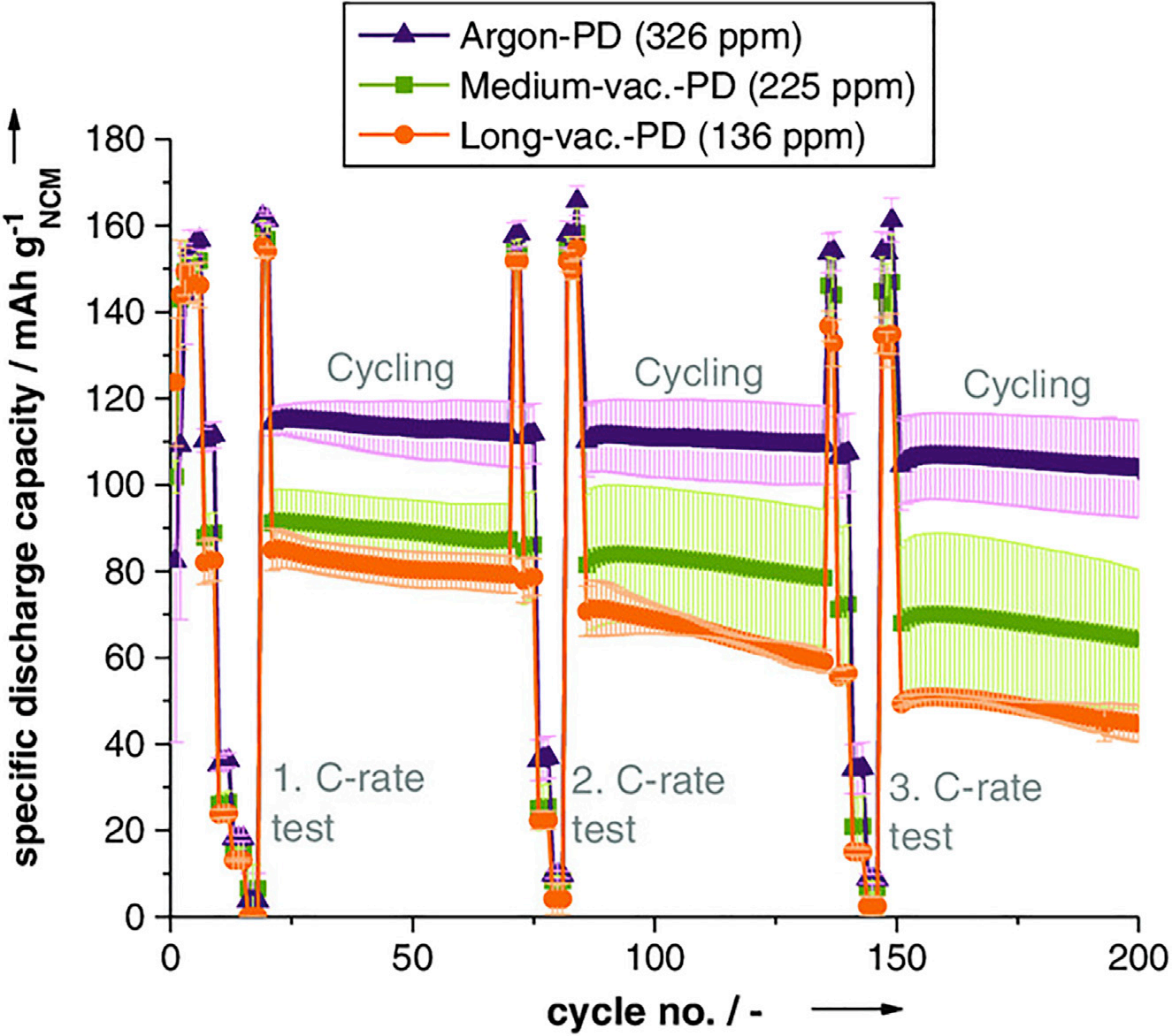
Impact of Post-Drying Procedure on Graphite-LiNi_0.6_Co_0.2_Mn_0.2_O_2_ Pouch Cell Discharge Capacity Specific discharge capacity of graphite-LiNi_0.6_Co_0.2_Mn_0.2_O_2_ pouch cells after various post-drying procedures. The sample that was not post-dried did not produce a viable cell. Reprinted with permission from Huttner et al. Energy Technology, Copywrite 2020 Wiley and Sons(Huttner et al., 2020).

Contrary to most of the data that are available for hydrated polymer electrolytes, these results showed that increasing water content does not result in increasing improvement of the electrochemical and mechanical performance of the electrode materials (Huttner et al., 2020). Battery cycling experiments showed that the cell assembly that was dried in argon only had the best long-term electrochemical performance and the best c-rate capacity (Figure 7) (Huttner et al., 2020). The electrochemical performance of the cell that was not post-dried could not be evaluated as the assembly of a pouch cell under atmospheric conditions produced a device that could not be cycled (Huttner et al., 2020).

The results of this experiment show that while care is taken during battery assembly to prevent water absorption and to remove water from the materials, the hygroscopic nature of many battery components can result in thousands of ppm of water being present in a cell when the contributions of each component are accounted for (Huttner et al., 2020). However, the majority of the water can be removed by simple post-drying procedures. Even though significant water content (1000 ppm ≤) was shown to decrease battery performance, the sample that was dried under argon (326 ppm H_2_O) outperformed the samples that were dried under vacuum (Huttner et al., 2020). These results suggest that while significant water content can degrade battery performance, water content on the order of several hundred ppm may benefit electrode performance.

As the study by Huttner et al. (2020) showed that the effects of absorbed water on electrode performance may be dependent on the quantity of water present, it is worth investigating what can happen to typical anode and cathode materials in lithium batteries upon exposure to different quantities of water. This has not been extensively studied in all-solid-state batteries, so this section will approach this question using data from systems that employ liquid electrolytes. In general, anodes tend to be more severely impacted by the presence of water than cathodes (Fu et al., 2005). This is because when water is absorbed on the surface of the graphite anodes, C-OH sites that disrupt the formation of the SEI layer can be formed (Fu et al., 2005). The formation of C-OH sites is an issue for battery performance because that they trap lithium ions upon intercalation resulting in irreversible capacity loss (Aurbach et al., 1999; Fu et al., 2005). In a system using a graphite anode, this was shown to result in an 11.5% capacity loss at 100 ppm water and a 22.2% capacity loss at 1000 ppm water (Fu et al., 2005). No significant impact was observed when water content was below 100 ppm in this system (Fu et al., 2005). These results suggest that total cell water content must remain quite low to minimize the impact on anode performance. Therefore, purposely adding water to the polymer electrolyte to improve ion mobility may impede battery performance by decreasing reversible capacity.

In addition to being sensitive to water, anode materials have been shown to be sensitive to other solvents such as acetonitrile which is commonly used in the preparation of PEO-based electrolytes. Trinh et al. (2018) expanded upon work by Rupich et al. (1982) who showed that exposing a lithium metal anode to an acetonitrile-containing electrolyte (LiPF_6_-acetonitrile) resulted in the degradation of the protective SEI layer and subsequent loss of capacity. This is a result of acetonitrile being thermodynamically unstable in the presence of the lithium metal (Rupich et al., 1982). In the study by (Trinh et al., 2018), pre-treating the lithium metal anode with fluoroethylene carbonate resulted in stable cycling in both half-cells and full cells employing LiFePO_4_ cathodes. Fluoroethylene carbonate pre-treatment resulted in the formation of an SEI layer comprised of LiF and lithium carbonates (Trinh et al., 2018).

Like electrolytes, many cathode materials are hygroscopic and can add several thousand ppm of water to a cell even with post-drying procedures in place. For example, in the Li-MnO_2_ cathode, 1-3 dioxolane electrolyte, lithium foil anode battery system that was studied by Aurbach et al. (1999), water in the hygroscopic cathode and electrolyte materials caused the SEI layer on the anode surface to break down resulting in a direct reaction between the lithium anode and water. This reaction was indicated by the presence of significant H_2_ gas evolution when the water content of the cell exceeded 1000 ppm (Aurbach et al., 1999). In addition to destroying the passivating layer on the anode, exposure to water and a lithium electrolyte can cause damage to the cathode by increasing the rate of aging and corrosion at the electrode surface (Stich et al., 2017). This process was measured electrochemically in a graphite-LiFePO_4_ coin cell with liquid electrolyte via the analysis of cycling stability and the build-up of new resistant phases as cell cycling progressed (Stich et al., 2017). The hygroscopic nature of the cathode materials makes controlling water content difficult as significant water re-absorption in LiFePO_4_ has been shown to occur within the first hour of exposure to moist air (Stich et al., 2017). The electrochemical performance of lithium cobalt oxide, another popular cathode material for use in lithium batteries, has also been shown to decrease as a result of surface degradation via water exposure. Exposure of the material to moisture has been linked to de-lithiation and decreased battery performance via the formation of poorly conducting species such as Li_2_CO_3_ and/or LiOH (Qian et al., 2018).

The impact of water absorption on the performance of NMC was studied via the preparation of NMC-PVDF cathodes by solvent casting in both water and NMP (Li et al., 2016). After primary drying, the cathode that was cast in water contained 625 ppm water which was decreased to 160 ppm after drying under vacuum at 100°C for two hours (Li et al., 2016). Cathode samples that were cast in NMP contained 200 ppm water after primary drying which was reduced to 87 ppm following drying under vacuum at 100°C for two hours (Li et al., 2016). In both cases, it was difficult to completely remove water from the cathode materials. In terms of electrochemical performance, half-cells prepared using cathodes that were dried at higher temperatures exhibited improved cycling efficiency as water decomposition during charging, which was shown to decrease battery efficiency, became less of an issue (Li et al., 2016). It was found that the total water content had a greater impact on cell performance than whether the cathode was initially prepared in water or NMP (Li et al., 2016).

## Conclusion

The analysis of solvated polymer electrolytes showed that adding water or other solvents to solid polymer electrolytes is commonly done inadvertently and tends to result in increased ion mobility and decreased polymer crystallinity. Although these modifications are typically associated with improved electrolyte performance, there is evidence suggesting that solvating the polymer electrolyte may not benefit the whole battery assembly. First, increases in measured ionic conductivity could include contributions from proton conductivity (Fullerton-Shirey and Maranas, 2009). Contributions to ionic conductivity from proton conduction are difficult to separate from those from lithium conduction without battery cycling and do not contribute to the electrochemical performance of a full cell. Additionally, electrode performance has been shown to become impaired under the presence of hundreds of ppm of water and become significantly degraded in the presence of thousands of ppm of water (Aurbach et al., 1999; Stich et al., 2017; Huttner et al., 2020). Despite the significant impact of water and/or solvent absorption on the electrolyte and electrodes in lithium batteries, its effects tend to be under-reported. This leaves researchers with many opportunities to measure, report, and better understand the role that water, and other solvents, can play in the workings of polymer electrolytes and all-solid-state batteries as a whole.

As a result of overall decreased battery performance in systems containing more than a couple hundred ppm solvent, the authors of this work recommend that researchers seek to minimize solvent content in all battery components during all processing steps. Since many electrolyte and cathode materials readily absorb solvents from atmosphere and casting media, it is suggested to avoid/reduce solvent use where possible. Some suggestions for doing so include employing dry processing techniques for electrolyte and electrode materials and capitalizing on solvent-free polymerization reactions to produce electrolytes and binders. In addition to reducing solvent use in processing, extensive drying procedures such as drying all materials twice and employing increased temperatures and vacuum techniques as well as storage of all materials in dry environments is recommended (Mankovsky et al., 2020).

Additionally, not measuring or reporting solvent content can make comparing various polymer electrolyte materials difficult as ionic conductivity can be significantly improved as a result of the presence of even several hundred ppm of absorbed solvent originating from the hygroscopic nature of polymers and/or lithium salts. It is therefore proposed that authors reporting on properties relating to the ionic conductivity and/or the polymer structure of solid polymer electrolytes should include measurements of the water/solvent content of their systems such that all potential mechanisms for the reported conductivities are considered and that experimental reproducibility can be improved.

## References

[bib1] Andrews W.T., Cook J., Marsh P., Ciocanel C., Lindberg G.E., Browder C.C. (2018). Development of a PEO-based lithium ion conductive epoxy resin polymer electrolyte. Solid State Ionics.

[bib2] Armstrong R.D., Clarke M.D. (1984). Lithium ion conducting polymeric electrolytes based on poly(ethylene adipate). Electrochimica Acta.

[bib3] Armstrong R.D., Clarke M.D. (1984). The effect of traces of water on PEO4.5LiCF3SO3 films. Solid State Ionics.

[bib4] Aurbach D., Zaban A., Dan P. (1999). On the role of water contamination in rechargeable Li batteries. Electrochimica Acta.

[bib5] Bhattacharja S., Smoot S.W., Whitmore D.H. (1986). Cation and anion diffusion in the amorphous phase of the polymer electrolyte (PEO)8LiCF3SO3. Solid State Ionics.

[bib7] Cheng X.B., Zhang R., Zhao C.-Z., Wei F., Zhang J.-G., Zhang Q. (2015). A review of solid electrolyte interphases on lithium metal anode. Adv. Sci..

[bib8] Cheng X.B., Zhang R., Zhao C.-Z., Zhang Q. (2017). Toward safe lithium metal anode in rechargeable batteries: a review. Chem. Rev..

[bib6] Chen-Yang Y.W., Chen H.C., Lin F.J., Chen C.C. (2002). Polyacrylonitrile electrolytes: 1. A novel high-conductivity composite polymer electrolyte based on PAN, LiClO4 and α-Al2O3. Solid State Ionics.

[bib9] Chintapalli M., Olson K.R., Mecham S.J., Devaux D., DeSimone J.M., Balsara N.P. (2016). Relationship between conductivity, ion diffusion, and transference number in perfluoropolyether electrolytes. Macromolecules.

[bib10] Commarieu B., Collin-Martin S., Gagnon C., Vijh A., Guerfi A., Zaghib K. (2019). Solid-to-liquid transition of polycarbonate solid electrolytes in Li-metal batteries. J. Power Sourc..

[bib11] Donoso J.P., Bonagamba T.J., Nascimento O.R., Panepucci H. (1995). Magnetic resonance study of water absorption in some peo-lithium salt polymer electrolytes. Electrochimica Acta.

[bib12] Dormidontova E.E. (2002). Role of competitive PEO-water and water-water hydrogen bonding in aqueous solution PEO behavior. Macromolecules.

[bib13] Forsyth M., Macfarlane D.R., Hill A.J. (2000). Compositional dependence of free volume in PAN/LiCF3SO3 polymer-in-salt electrolytes and the effect on ionic conductivity. J. Polym. Sci. B Polym. Phys..

[bib14] Forsyth M., Jiazeng S., MacFarlane D.R. (2000). Novel high salt content polymer electrolytes based on high Tg polymers. Electrochimica Acta.

[bib15] Fu L.J., Wu Y.P., Wu H.Q., Holze R. (2005). Surface active sites: an important factor affecting the sensitivity of carbon anode material toward humidity. Electrochem. Solid-state Lett..

[bib16] Fullerton-Shirey S.K., Maranas J.K. (2009). Effect of LiClO 4 on the structure and mobility of PEO-based solid polymer electrolytes. Macromolecules.

[bib17] Hakem I.F., Lal J., Bockstaller M.R. (2006). Mixed solvent effect on lithium-coordination to poly(ethylene oxide). J. Polym. Sci. B Polym. Phys..

[bib18] Harris C.S., Rukavina T.G. (1995). Lithium ion conductors and proton conductors: effects of plasticizers and hydration. Electrochimica Acta.

[bib19] Homann G., Nair J., Laskovic I.C., Winter M., Kasnatscheew J. (2020). Poly(Ethylene oxide)-based electrolyte for solid-state-lithium-batteries with high voltage positive electrodes: evaluating the role of electrolyte oxidation in rapid cell failure. Sci. Rep..

[bib20] Husken D., Gaymans R.J. (2008). The structure of water in PEO-based segmented block copolymers and its effect on transition temperatures. Macromol. Chem. Phys..

[bib21] Huttner F., Haselrieder W., Kwade A. (2020). The influence of different post-drying procedures on remaining water content and physical and electrochemical properties of lithium-ion batteries. Energy Technol..

[bib22] Johansson A., Lauenstein A., Tegenfeldt J. (1995). Effect of water on diffusion and ionic conductivity in PEG and LiCF3SO3PEG10. J. Phys. Chem..

[bib23] Kao, Chao S.-W., Lee C.-H. (2006). 7Li NMR, ionic conductivity and self-diffusion coefficients of lithium ion and solvent of plasticized organic-inorganic hybrid electrolyte based on PPG-PEG-PPG diamine and alkoxysilanes. Electrochimica Acta.

[bib24] Kerr R., Arthur T.S., Pathirana T., Mizuno F., Takechi K., Forsyth M., Howlett P.C. (2018). Water-tolerant lithium metal cycling in high lithium concentration phosphonium-based ionic liquid electrolytes. Sustainable Energy Fuels.

[bib25] Kimura K., Panero S., Scrosati B., Tominaga Y. (2015). Electrochemical properties of a poly(ethylene carbonate)-LiTFSI electrolyte containing a pyrrolidinium-based ionic liquid. Ionics.

[bib60] Łatoszyńska A.A., Taberna Pierre Louis, Simon Patrice, Wieczorek Władysław (2017). Proton conducting gel polymer electrolytes for supercapacitor applications. Electrochimica Acta.

[bib26] Li J., An S.J., Wood D. (2016). Evaluation residual moisture in lithium-ion battery electrodes and its effect on electrode performance. MRS Adv..

[bib27] Liu W., Lin D., Shi F., Wang S., Sendek A.D., Cui Y. (2017). Enhancing ionic conductivity in composite polymer electrolytes with well-aligned ceramic nanowires. Nat. Energy.

[bib28] Ma X., Yu J., He K. (2006). Thermoplastic starch plasticized by glycerol as solid polymer electrolytes. Macromol. Mater. Eng..

[bib29] MacFarlane D.R., Zhou F., Forsyth M. (1998). Ion conductivity in amorphous polymer/salt mixtures. Solid State Ionics.

[bib30] Mankovsky D., Lachal M., Caradant L., Aymé-Perrot D., Dollé M. (2020). Water content in solid polymer electrolytes: the lost knowledge. Chem. Commun..

[bib31] Mindemark J., Bowden T., Brandell D. (2018). Beyond PEO-Alternative host materials for Li + -conducting solid polymer electrolytes. Prog. Polym. Sci..

[bib32] Ohno S., Buchheim J., Duchardt M., Hatz A.-K., Kraft M.A., Kwak H., Santhosha A.L., Liu Z., Minafra N., Tsuji F. (2020). How certain are the reported ionic conductivities of thiophosphate-based solid electrolytes? An interlaboratory study. ACS Energy Lett..

[bib33] Peled E., Menkin S. (2017). ‘Review—SEI: past, present and future’. J. Electrochem. Soc..

[bib34] Qian J., Yang J., Li S., Wang X., Zhuang H.L., Lu Y. (2018). Electrochemical surface passivation of LiCoO2 particles at ultrahigh voltage and its applications in lithium-based batteries. Nat. Commun..

[bib35] Rubin J., Andrews R.D. (1968). Effect of solvent treatments on the mechanical properties of nylon 6. Polym. Eng. Sci..

[bib36] Rupich M.W., Pitss L., Abraham K.M. (1982). ‘Characterization of reactions and products of the discharge and forced overdischarge of Li∕SO[sub 2] cells’. J. Electrochem. Soc..

[bib37] Sepe M. (2014). Why (And what) You Need to Dry, Plastics Technology. https://www.ptonline.com/articles/why-and-what-you-need-to-dry.

[bib39] Stich M., Pandey N., Bund A. (2017). Drying and moisture resorption behaviour of various electrode materials and separators for lithium-ion batteries. J. Power Sourc..

[bib40] Sun B., Edström K., Brandell D. (2014). Polycarbonate-based solid polymer electrolytes for Li-ion batteries. Solid State Ionics.

[bib41] Sun B., Mindemark J., Gustafsson T., Edström K., Brandell D. (2015). At the polymer electrolyte interfaces: the role of the polymer host in interphase layer formation in Li-batteries. J. Mater. Chem. A..

[bib42] Tanzella F.L., Frydrych D., Farrington G.C., Story H.S. (1981). Ion transport in peo-alkali salt complex polymeric electrolytes. Solid State Ionics.

[bib43] Tominaga Y., Asai S., Sumita M. (2009). Effect of humidity on ionic conductivity of NBR/polyether electrolyte blends with microscale Sea-Island phase separation. Nippon Gomu Kyokaishi.

[bib44] Trinh N.D., Aymé-Perrot D., Badia A., Dollé M., Rochefort D. (2018). An artificial lithium protective layer that enables the use of acetonitrile-based electrolytes in lithium metal batteries. Angew. Chem. Int. Ed..

[bib45] Verdier N., Zidani R., Prébé A., Aymé-Perrot D., Pellerin C., Dollé M., Rochefort D. (2020). Crosslinked polyacrylonitrile-based elastomer used as gel polymer electrolyte in Li-ion battery applications. Appl. Energy Mater..

[bib46] Voigt N., Van Wüllen L. (2012). The mechanism of ionic transport in PAN-based solid polymer electrolytes. Solid State Ionics.

[bib47] Voigt N., Van Wüllen L. (2014). The effect of plastic-crystalline succinonitrile on the electrolyte system PEO:LiBF4: insights from solid state NMR. Solid State Ionics.

[bib48] Wang Z., Huang H., Chen L., Xue R., Wang F. (1996). Investigation of the position of Li+ ions in a polyacrylonitrile-based electrolyte by Raman and infrared spectroscopy. Electrochimica Acta.

[bib49] Wang Z., Huang X., Mo Y., Chen L. (2001). Ion transport polyacrylonitrile-based electrolytes with high LiTFSI contents. Electrochem. Solid-state Lett..

[bib50] Wang Z., Chen L., Mo Y., Huang X. (2002). Raman and AC impedance spectroscopic studies on roles of polyacrylonitrile in polymer electrolytes. J. Electrochem. Soc..

[bib51] Wang A., Li H., Shi S., Qi Y. (2018). Review on modeling of the anode solid electrolyte interphase (SEI) for lithium-ion batteries. Npj Comput. Mater..

[bib38] Wegner G. (2006). Polymers as functional components in batteries and fuel cells. Polym. Adv. Tech..

[bib52] Weston J.E., Steele B.C.H. (1982). Effects of preparation method on properties of lithium salt-poly(ethylene oxide) polymer electrolytes. Solid State Ionics.

[bib53] Wu Q.Y., Chen Xiao-Na, Wan Ling-Shu, Xu Zhi-Kang (2012). Interactions between polyacrylonitrile and solvents: density functional theory study and two-dimensional infrared correlation analysis. J. Phys. Chem. B.

[bib54] Yang Y., Huo H. (2013). Investigation of structures of PEO-MgCl2 based solid polymer electrolytes. J. Polym. Sci. Part B: Polym. Phys..

[bib55] Yushkin A.A., Vasilev A.A., Karpacheva G.P., Volkov A.V. (2018). PAN filtration membranes with extended solvent stability. J. Phys. Conf. Ser..

[bib56] Zhang Z., Ohl Michael, Diallo Souleymane O., Jalarvo Niina H., Hong Kunlun, Han Youngkyu, Smith Gregory S., Do Changwoo (2015). Dynamics of water associated with lithium ions distributed in polyethylene oxide. Phys. Rev. Lett..

[bib57] Zhang X., Wang Y., Zhou Q. (2018). Influence of humidity on the complex structure of PEO-lithium salt polymer electrolyte. Polym. Sci. Ser. A.

[bib58] Zhou C., Bagau S., Lv B., Thangadurai V. (2020). Understanding the role of solvents on the morphological structure and Li-ion conductivity of poly(vinylidene fluoride)-based polymer electrolytes. J. Electrochem. Soc..

[bib59] Łasińska A.K., Marzantowicz M., Dygas J.R., Krok F., Florjańczyk Z., Tomaszewska A., Zygadło-Monikowska E., Zukowska Z., Lafont U. (2015). Study of ageing effects in polymer-in-salt electrolytes based on poly(acrylonitrile-co-butyl acrylate) and lithium salts. Electrochimica Acta.

